# Improved Morphological Filter Based on Variational Mode Decomposition for MEMS Gyroscope De-Noising

**DOI:** 10.3390/mi9050246

**Published:** 2018-05-17

**Authors:** Yicheng Wu, Chong Shen, Huiliang Cao, Xu Che

**Affiliations:** 1 National Key Laboratory of Electronic Measurement Technology, North University of China, Shanxi 030051, China; 1501054235@st.nuc.edu.cn (Y.W.); 1506024118@st.nuc.edu.cn (X.C.); 2 College of Mechatronics Engineering, North University of China, Shanxi 030051, China; 3 School of Instrument and Electronics, North University of China, Shanxi 030051, China

**Keywords:** MEMS gyroscope, variational mode decomposition, morphological filter, denoising algorithm

## Abstract

An adaptive multi-scale method based on the combination generalized morphological filter (CGMF) is presented for de-noising of the output signal from a MEMS gyroscope. A variational mode decomposition is employed to decompose the original signal into multi-scale modes. After choosing a length selection for the structure element (SE), the adaptive multi-scale CGMF method reduces the noise corresponding to the different modes, after which a reconstruction of the de-noised signal is obtained. From an analysis of the effect of de-noising, the main advantages of the present method are that it: (i) effectively overcomes deficiencies arising from data deviation compared with conventional morphological filters (MFs); (ii) effectively targets the different components of noise and provides efficacy in de-noising, not only primarily eliminating noise but also smoothing the waveform; and (iii) solves the problem of SE-length selection for a MF and produces feasible formulae of indicators such as the power spectral entropy and root mean square error for mode evaluations. Compared with the other current signal processing methods, the method proposed owns a simpler construction with a reasonable complexity, and it can offer better noise suppression effect. Experiments demonstrate the applicability and feasibility of the de-noising algorithm.

## 1. Introduction

Because of its unique properties, the gyroscope has been widely used in navigation, aviation, aerospace and the national economy, which is one of the key development technologies. Fiber optic gyroscope (FOG) is a rotation sensor with the advantages of long life, dynamic range and short start-up time. The resonator fiber optic gyroscope (RFOG) has an improved shot noise limited signal to noise sensitivity for a given size. Qiu et al. [1] proposed a bench-top RFOG using external-cavity laser stabilization and optical filtering, performing well toward navigation grade. Fsaifes et al. [2] investigated on a 19-cell hollow-core photonic-bandgap fiber resonator for rotation sensing applications by using a simple-bulk cavity design. Resonant micro optical gyros (RMOGs) are considered ideal candidates for optoelectronic gyroscope miniaturization, whose prototypes are complexity modules including several packaged optoelectronics components connected by optical fibers. Dell’Olio et al. [3] finished design, fabrication and initial characterization of large-size InGaAsP/InP ring resonators for gyroscope applications. Their cavity Q-factor (= 74,000 ± 20%) can realize at least six times larger than the state-of-the-art. Liang et al. [4] studied resonant micro photonic gyroscopes, and demonstrated a high-performance gyroscope based on a high-Q whispering gallery mode resonators (WGMRs). However, the FOG precision control of the instrument presents issues of high cost and bulky size. MEMS gyroscopes are a kind of angular sensor, which has seen good development in recent decades. In this paper, we are devoted to researching the dual-mass decoupled MEMS gyroscope.

The MEMS gyroscope appears in many different fields for application, such as automobiles, consumer electronics, and robotics, as it is relatively smaller in size and lower in weight, power consumption, and price. High-precision MEMS-based inertial gyroscopes reported in the literature have bias drift values that are better than the tactical grade performance requirements. Noise contained in the original signal of these low-cost gyroscopes degrades its accuracy and limits its applications. Consequently, signal noise reduction is essential to enhance their performance [5,6,7,8,9]. Kalman filter (KF) is a representative algorithm for gyroscope de-noising for practical inertial navigation and integrated navigation application [10], however, the filter models and noise characteristics will influence the performance easily [11].

The morphological filter (MF) is a nonlinear time-frequency analysis method capable of extracting local features and eliminating instantaneous impulses [12]. Various researchers have introduced variants of the MF. Lv et al. [13] introduced average a combination difference morphological filter for feature extraction, where the scale selection of structural elements (SEs) is determined by the Teager energy kurtosis (TEK). Hu et al. [14] proposed a new improved MF algorithm to overcome the deficiency of conventional MFs which can be easily interfered with. In [15], a combination of a MF and translation-invariant wavelet decomposition was employed to augment ensemble empirical mode decomposition (EEMD) and to improve de-noising reliability. Li et al. [16] used multi-scale MFs (MSMFs) in gear fault diagnostics and verified the averaged multiscale morphological dilate-erode gradient (AMMGDE) filter performs the best. In [17], a MF was used for field applications of on-line monitoring and diagnosis to prevent misjudgments in detection.

Indeed, the MF is capable of directly extracting the geometric structure of an impulsive feature in comparison with other non-stationary signal processing methods, such as the fast Fourier Transform (FFT), the wavelet transform, the Hilbert-Huang transform (HHT) and the empirical mode decomposition (EMD) [18,19,20], These methods are limited by their respective drawbacks. The FFT does not conform well to transient and non-stationary signals processing. Short-time FFT is unable to determine a choice of window size in regard to the frequency requirement constraint of random signals [21]. The HHT requires an appropriate selection of basic functions to avoid faults diagnosed using the intrinsic mode functions (IMFs) [22]. In this regard, MF excels in accuracy and extensive applicability. Nevertheless, conventional MF also has its own limitations. Morphological operators generally suffer from different output biases and SE selection problems [23]. Selecting a key scale for the SE requires a prior knowledge from multiple attempts. The multiple frequency components of high-intensity noise cannot be completely smoothed [24]. To overcome the above deficiencies, the variational mode decomposition (VMD) is a non-recursive time-frequency method for non-stationary analyses. Its employment effectively accomplishes a signal-adapted decomposition to provide signal-noise separation. The decomposed signal component is more compatible with processing conditions of a MF. For modifying methods combination, an optimized synthetical promote method is presented [19,25,26].

To overcome the limitation of the MF in handling measurement noise, particularly in the application to signal de-noising of a gyroscope, we propose an adaptive multi-scale combination generalized morphological filter (CGMF) based on the VMD method. The assisting algorithm has a strong capability to extract and eliminate noise. Using a preliminary waveform and a frequency spectrogram analysis of the raw signal, a SE with specific properties is selected to suppress noise related to the specific attribute. The elimination of the dominant noise obtained demonstrates that the proposed multi-scale CGMF algorithm is quite suitable. Nevertheless, choosing an optimal length for the SE is a current focus of attention and researchers have developed various guidelines for this choice. Through multitudinous data analyses, feasible regulatory formulae providing indicator values to summarize data is given for assessments. The formulae adopt the power spectral entropy (PSE) and root mean square error (RMSE) to evaluate the efficacy of the sampling SEs chosen. Hence, SE selection and the critical choice of scale for SE are reasonably settled within this CGMF-VMD scheme. Moreover, the application of VMD can decompose a raw signal into band-limited intrinsic mode functions (BLIMFs) [27]. Through hierarchical processing, the high and low frequency domains are respectively denoised by the optimized CGMF. The proposed method overcomes a statistical bias problem of the MF, sets selection criteria for the length choice, and remedies the deficiencies in MF high-intensity noise elimination and the excessive mode eliminations that lead to distortions in VMD analysis.

## 2. Adaptive Multi-Scale CGMF Combined with the VMD

### 2.1. Theories of the CGMF

Georges Matheron and Jean Serra proposed the notion of mathematical morphology based on integral geometry [28]. Developed to be applied for signal proceeding, the mathematical MF is a robust one-dimensional nonlinear signal processing based on set theory. The key concept of MF is to modify the local characteristics of the signal through its interaction by a predefined SE, which slides through points in the signal and modifies the geometric shape of the signal. The MF performs better than linear filters in noise reduction and in identifying chief morphological characteristics.

In MF analysis, four basic operators arise: dilation, erosion, opening, and closing denoted ⊕, Θ, •, and ∘ respectively. The opening and closing operators are constructed based on the dilation and erosion operators. The basic operators form the basis of a morphological method and defined as follows:

Dilation:

(1) $$(f⊕b)(x)=\underset{\begin{array}{l} m-0,1\ldots ,m-1\\ x-0,1\ldots ,x-m \end{array}}{\max}\left\{f(x-m)+b(m)\right\},$$

Erosion:

(2) $$(f\Theta b)(x)=\underset{\begin{array}{l} m-0,1\ldots ,m-1\\ x-0,1\ldots ,x+m-2 \end{array}}{\min}\left\{f(x+m)-b(m)\right\},$$

Closing:

(3) (f•b)(x)=[(f⊕b)Θb](x),

Opening:

(4) (f∘b)(x)=[(fΘb)⊕b](x).

where *f*(*x*) is the original one-dimensional discrete signal, a function over a definition domain *F* = {0, 1, … *N* − 1}; B(x) the SE, defined on set *B* = {0,1, … *M −* 1} (*M > N*); *X* a sample in the signal; and *M* a point in SE.

The morphological close-opening gradient filter ($F_{oc}$) and the open-closing gradient filter ($F_{co}$) are defined by combinations of the above operations,

(5) $$F_{oc}\left(f\left(x\right)\right)=\left(f\circ b•b\right)\left(x\right),$$

(6) $$F_{co}\left(f\left(x\right)\right)=\left(f•b\circ b\right)\left(x\right)$$

Dilation smooths positive peaks and erosion fills up negative ones. The opening operation suppresses the positive impulses; the closing operation is applied to suppress the negative impulses [29]. In view of the attributes of the opening operator’s expansibility and the closing operator’s contractibility, the output magnitude of $F_{oc}$ and $F_{co}$ becomes small and large, respectively. Thus, to overcome the statistical bias and boost the de-noising capability, an average weighted combination of the close-opening and open-closing operations is adopted,

(7) $$h\left(x\right)=\psi_{oc\left(co\right)}\left(b\right)=\frac{F_{co}\left(f\left(x\right)\right)+F_{oc}\left(f\left(x\right)\right)}{2}.$$

where *h*(*x*) corresponds to the processed signal, and $\psi_{oc(co)}\left(b\right)$ denotes the basic operations unit of the MF.

Whereas the SEs significantly affect the effectiveness of the MF, the raw signal always incorporates heavy irregular noise. The singular nature of the SE generally favors a single type of noise. To produce faster performances in denoising and preserving, a combination of diverse SEs combined with the open-closing and close-opening operators is adopted to establish the CGMF.

Based on Equations (5) and (6), the combined SEs of the morphological close-opening gradient filter ($G_{co}$) and the open-closing gradient filter ($G_{oc}$) are defined as

(8) $$G_{oc}\left(f\left(x\right)\right)=\left(f\circ b_{1}•b_{2}\right)\left(x\right),$$

(9) $$G_{co}\left(f\left(x\right)\right)=\left(f•b_{2}\circ b_{1}\right)\left(x\right).$$

Using Equation (7), the average weighted combination of the close-opening and open-closing operations of the CGMF is defined as

(10) $$y\left(x\right)=\psi_{G_{oc}\left(G_{co}\right)}\left(b_{1},b_{2}\right)=\frac{\left[G_{oc}\left(f\left(x\right)\right)+G_{co}\left(f\left(x\right)\right)\right]}{2}$$

The CGMF is capable of eliminating large-scale disturbances from heavy noise and is effective in restoring the original signal features. Also, the statistical bias is suppressed appropriately. Indeed, de-noising using CGMF is discernibly superior in comparison with a single SE MF. A table for CGMF algorithm (Algorithm 1) is introduced in detail.

### 2.2. Influence Parameters about MF

SE has a drawback affecting the effectiveness and accuracy of the MF. The types of SE are various and include the triangular SE (TSE), the sinusoidal SE, and the semicircular SE (SSE) [30]. The corresponding geometrical characteristic of the SE effectively determines the de-noising capability when targeting a specific type of noise. As confirmed by the numerous valid data, the TSE appears to be quite appropriate for detecting impulsive noise; SSE effectively eliminates Gaussian white noise. Hence, with this article focused on an analysis of nonlinear signal suppression, the TSE and SSE are adopted in constructing the CGMF.

For example, a sinusoidal signal mixed with an impulse η and Gaussian noise φ has the form

(11) h(t)=3sin(2πt)+5cos(6πt)+φ+η

**Figure 1:**
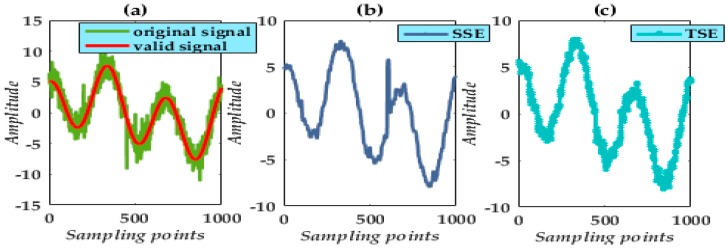
Signal processing by different SE: (**a**) original signal; (**b**) single SSE; (**c**) single TSE.

A conventional MF method was applied to reduce the signal noise using a single SSE and TSE. A comparison of results is shown in Figure 1. The experiment demonstrates that the resultant waveforms are different. The waveforms reflect the specific noise eliminated. That is, SE selection must be done cautiously.

In general, shape, height (amplitude), and length (domain) are three significant elements determining the attributes of the SEs. With validated statistical analysis, the SE shape should match well the signal. The height should be appropriate, not extra-small or extra-large. In some instances, the height may be irregular and play a lesser role relative to the SE length. Thus, length is also paramount in filter design and depends on specialized de-noising features of the SEs and characteristics of the input signal.

**Figure 2:**
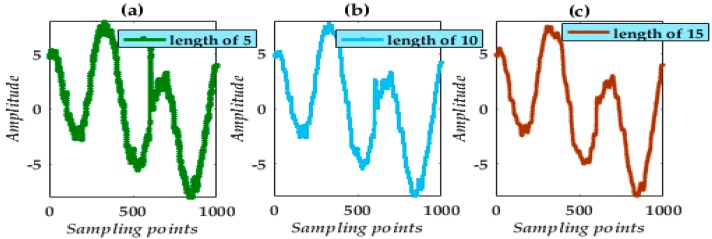
De-noising waveforms using a single SSE with lengths of 5, 10, and 15.

**Figure 3:**
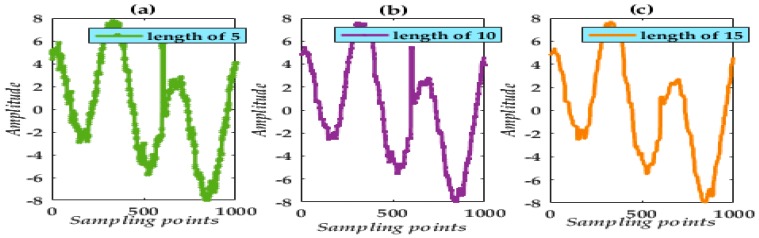
De-noising waveforms using a single TSE with lengths of 5, 10, and 15.

Figure 2 and Figure 3 show the results following processing using a single SSE MF and single TSE MF and a selection of lengths.

The results obtained indicate that noise suppression critically depends on SE choice and length. In addition, there exists a problem with a strong deviation in the MF processing. Simultaneously, from the waveform analysis, noise suppression is impracticable. To achieve a more practical noise reduction in the final processed result, VMD appears impressive.

As a recent non-recursive signal processing technique, VMD adaptively decomposes the real-valued signal into a series of modes from high to low frequencies. It also performs better in preserving amplitude and reducing random noise compared with, for example, EMD and wavelet analysis.

The unprocessed signal is often heavily mixed with multi-variable spectral-domain signals immersed in noise of different intensities and frequencies. Hence VMD decomposes the multi-component signal into several intrinsic mode functions (IMFs), which represent the fundamental oscillatory modes of the signal, each being compact around a center pulsation. Initially the decomposition does a preliminary de-noising treatment of the composite signal. The decomposed modes are specific to a single spectral component with stabilized features and lower disturbances, and designed to lower the complexity of the spectral analysis. Thus, the VMD application primarily ensures accuracy with the allusion of heavy noise elimination.

**Algorithm 1:** CGMF algorithm.**SE construction:** Generate a pixel pitch *P* of SEs Input row data *df*Estimate a priori-knowledge SE length *N*
**for** i = 1, …, *N*
**do** assign amplitude variable NHOD (i), OSE (i) and NH (i) **end for**Construct triangular SE: $b_{1}$=strel (‘arbitrary’, NHOD (i), OSE (i)) Construct semicircular SE: $b_{2}$=strel (‘arbitrary’, NHOD (i), NH (i)) **Operators combination:** Construct closing and opening operators using Equations (3) and (4) Construct gradient filter $G_{co}$ and gradient filter $G_{oc}$ using Equations (8) and (9) Compute average weighted combination $\psi_{G_{oc}\left(G_{co}\right)}$ using Equation (10)

### 2.3. Implementing the VMD Theory

While a recently developed technique by Dragomiretskiy and Zosso (2014) for adaptive signal decomposition, VMD has been effectively applied to extract instantaneous time-frequency features from non-stationary signals. It can non-recursively decompose a nonlinear multi-component signal f(t) into a discrete number of quasi-orthogonal BLIMFs, $u_{k}\left(k\in 1,2,3\ldots k\right)$, with specific sparsity properties of its bandwidth in the spectral domain. Also, each mode is compact around a center frequency $w_{k}\left(k\in 1,2,3\ldots k\right)$. The fundamental problem for the VMD is to solve the constrained variational problem; i.e., $H^{1}$ the Gaussian smoothness is used to estimate the bandwidth of $w_{k}$ [31]. The variational problem is mathematically expressed in the form

(12) $$\underset{\left\{u_{k},w_{k}\right\}}{min}\left\{\sum_{k=1}^{k}{\left\|\partial_{t}\left[\left(\partial \left(t\right)+\frac{j}{\pi t}\right)\times u_{k}\left(t\right)\right]e^{-jwk^{t}}\right\|}_{2}^{2}\right\},$$

(13) $$\sum_{k=1}^{k}u_{k}=f\left(t\right),$$

where f(t) is the original valid signal, k denotes the number of modes, and *t* represents elapsed time.

With the objective of converting the constrained variational problem to an unconstrained one, a quadratic penalty term and Lagrangian multiplier are used. The quadratic penalty term ensures high precision of the signal reconstruction even under instances of strong noise. The Lagrangian multiplier make the constraint condition retains stringency. The equation for the augmented Lagrangian L is

(14) $$L\left(u_{k},w_{k},\lambda \right)=\alpha {\sum_{k=1}^{k}\left\|\partial_{t}\left[\left(\partial \left(t\right)+\frac{j}{\pi t}\right)u_{k}\left(t\right)\right]e^{-jwk^{t}}\right\|}_{2}^{2}+{\left\|f\left(t\right)-\sum_{k=1}^{k}u_{k}\left(t\right)\right\|}_{2}^{2}+\langle \lambda \left(t\right),f\left(t\right)-\sum_{k=1}^{k}u_{k}\left(t\right)\rangle$$

where ∂ is the balancing parameter of the data-fidelity constraint, and λ denotes the Lagrange factor.

Next, the alternate direction method of multipliers enables the saddle point of the augmented Lagrangian to be found in a sequence of iterative sub-optimizations. The modes in the Fourier domain are updated essentially by Wiener filtering using a filter tuned to the current center frequency on the positive part of the spectrum with an integral form. The mode ${u_{k}}^{n+1}$ in the time domain is

(15) u^kn+1(ω)=f^(ω)−∑i≠ku^i(ω)+(λ^ω2)1+2α(ω−ωκ)2.

Wiener filtering is embedded in the VMD, and the center frequency $\omega_{k}$ is accordingly updated by ${\hat{u}}_{k}^{n+1}$. Equation (15) for ${\hat{\omega }}_{k}^{n+1}$, the optimization of which also takes place in the Fourier domain,

(16) $$\omega_{k}^{n+1}=\frac{\int_{0}^{\infty }\omega {\left|{\hat{u}}_{k}\left(\omega \right)\right|}^{2}d_{\omega }}{\int_{0}^{\infty }{\left|{\hat{u}}_{k}\left(\omega \right)\right|}^{2}d_{\omega }}$$

where ${\hat{u}}_{i}\left(w\right)$, ${\hat{u}}_{k}^{n+1}\left(w\right)$, $\hat{\lambda }\left(\omega \right)$, and $\hat{f}\left(\omega \right)$ denote the Fourier transforms of $u_{i}\left(\omega \right)$, $u_{k}^{n+1}\left(\omega \right)$, λ(ω), andf(ω), respectively, and n indexes the number of iterations.

### 2.4. The Proposed Multi-Scale Adaptive CGMF Combined with the VMD

Different SE characteristics match with real-time signal features differently; this hold for every given SE length also [32]. Adopting a self-adaptive CGMF with a corresponding SE combination and integrating with specific noise characteristics, the processed result demonstrates that this filter is more effective and accurate. In general, an excessively long length easily leads to over-treated distortions whereas an excessively small length is incapable of ensuring better noise extraction. Furthermore, the length choice of every SE should be normalized.

To evaluate the treatment performance, PSE and RMSE are adopted, which correlate well with the instantaneous disposal performance. Also, PSE is related to information entropy, which is able to quantify the spectral complexity of the unprocessed signal [33]. Here a brief introduction of PSE is given:

- **Power spectral entropy (PSE)**

PSE is an extension of Shannon entropy in the frequency domain and is linked to the distribution of frequency components [34]. The steps to obtain PSE are:

(1) Derive the power spectral calculation formula from Equation (16):
  (17) $$S\left(\omega \right)=\frac{1}{2\pi N}{\left|X\left(\omega \right)\right|}^{2}$$
  where *N* is the length of f(t), and X(ω) is the processed result obtained using FFT.
(2) According to the energy conservation law, the spectrum density function can be obtained using the normalization of the frequency components:
  (18) $$\sum x^{2}\left(t\right)\Delta t={\sum \left|X\left(\omega \right)\right|}^{2}\Delta \omega ,$$
(3) The power spectral entropy is defined as
  (19) $$\mathrm{PSE}=-\sum_{i=1}^{N}P_{i}\mathrm{lg}P_{i},$$
  where $P_{i}=\frac{s_{i}}{\sum_{i=1}^{N}s_{i}}$ denotes the specific gravity of the *i*-th sub-band spectral value over the whole power spectrum. The RMSE is defined as
  (20) $$\mathrm{RMSE}=\sqrt{{\left(\frac{1}{N}\sum_{t-1}^{N}x\left(t\right)-\bar{x}\left(t\right)\right)}^{2}}.$$

As comprehensive indicators, the PSE and RMSE values represent the degree of irregularity associated with the composite signal. Smaller values of these indices signify smoother shapes, implying that the signal is in a more ordered condition.

Frequently, overly long or short SE lengths lead easily to distortions and an under-curing problem of the composite signal results. Nevertheless, a given regulatory formula can overcome blindness from the empirical selection. The data formulae alluded to regarding the SEs are:

- Conclusively formulae for length determination

(1) TSE dimension selection:
  (21) $$length\left(u_{i+1}\right)=length\left(u_{i}\right)\cdot {\left(\frac{\mathrm{RMSE}\left(u_{i+1}\right)}{\mathrm{RMSE}\left(u_{i}\right)}\right)}^{2},$$
(2) SSE dimension selection:
  (22) $$length\left(u_{i+1}\right)=length\left(u_{i}\right)\cdot {\left(\frac{\mathrm{PSE}\left(u_{i+1}\right)}{\mathrm{PSE}\left(u_{i}\right)}\right)}^{2}.$$

**Figure 4:**
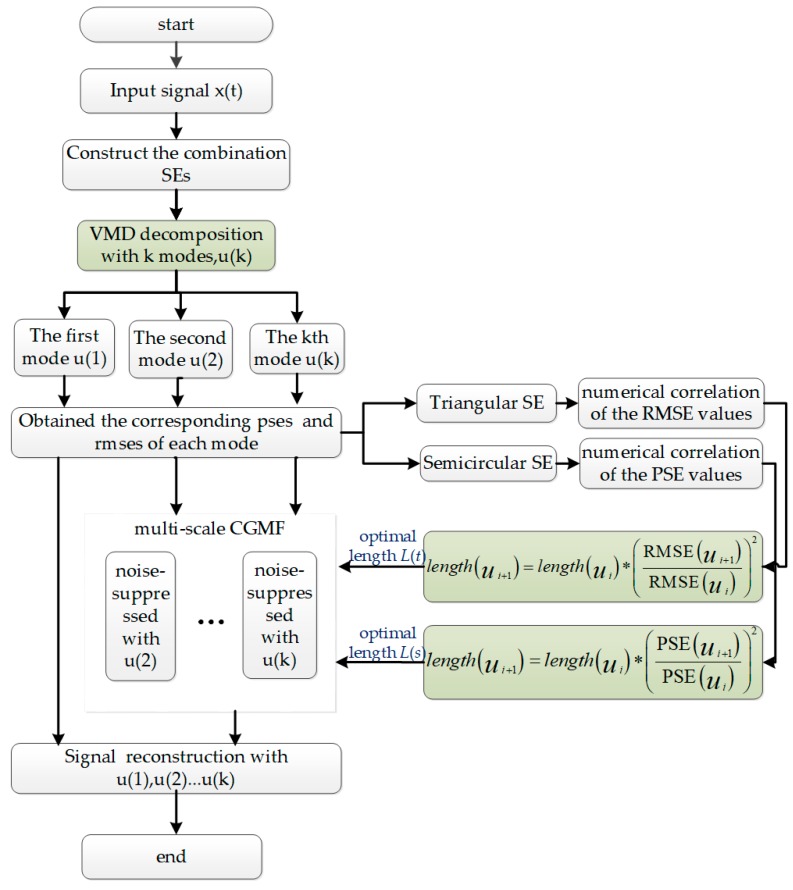
Flowchart of the proposed noise elimination method.

Given the determined length, from non-recursive signal processing, a series of modes are obtained. The high-order modes represent fast oscillations, which mainly are high-frequency contamination noise. With their extraction using VMD, the remaining low frequency modes are regarded as constituting the valid signal and need preserving. Among the high-order modes, to identify valid signal components mixed in with the noise, the adaptive multi-scale CGMF is used with corresponding calculated lengths to perform mode purification. Finally, the reconstructed signal contains the original low-frequency mode and treated high-frequency modes. A table for improved CGMF algorithm combined with VMD (Algorithm 2) is introduced in detail. Figure 4 shows the actual flowchart of the algorithm for the entire proposed method.

As the number of decomposed modes increase, the low-order modes may undergo waveform distortion. The proposed method can restore the whole valid signal to a large degree.

**Algorithm 2:** improved CGMF algorithm combined with VMD.**VMD decomposition:** Input row data *df* Select a decomposition mode value *k* Generate decomposed *k* modes $u_{k}$ **SE construction:** Run Algorithm 1 to generate pixel pitch *P* **for** i = 1, …, *N*
**do** assign amplitude variable NHOD (i), OSE (i) and NH (i) **end for** Construct TSE: $b_{1}$=strel (‘arbitrary’, NHOD (i), OSE (i)) Construct SSE: $b_{2}$=strel (‘arbitrary’, NHOD (i), NH (i)) **SE length determination:**Compute PSE and RMSE values of $u_{k}$ using Equations (17)–(19) Assign index PSE → Gaussian white noise *ϕ* → SSE Assign index RMSE → impulse noise *η*
**→** TSE Compute corresponding SE length N using Equations (21) and (22) **Operators combination:** Construct closing and opening operators using Equations (3) and (4) Construct gradient filter $G_{co}$ and gradient filter $G_{oc}$ using Equations (8) and (9) Compute average weighted combination $\psi_{G_{oc}\left(G_{co}\right)}$ using Equation (10) Estimate reconstruction signal Y=$\sum u_{k}{}^{\prime }$


## 3. Simulation Signal Analysis

### 3.1. Simulated Sinusoidal Signal

The original signal incorporates impulsive components and random Gaussian white noise commonly corrupting the whole signal. To verify the noise reduction and demodulation capability of the proposed method, a simulated signal contaminated by additive noise is generated for analysis,

(23) $$f\left(t\right)=e^{3t}\cdot \sin\left(3t\right)+\eta \left(t\right)+\varphi \left(t\right),$$

where φ(t) is the random Gaussian noise of intensity 1, and η(t) denotes the impulsive noise. The data length is 1000 for each sample. The sampling frequency is 1024 Hz.

**Figure 5:**
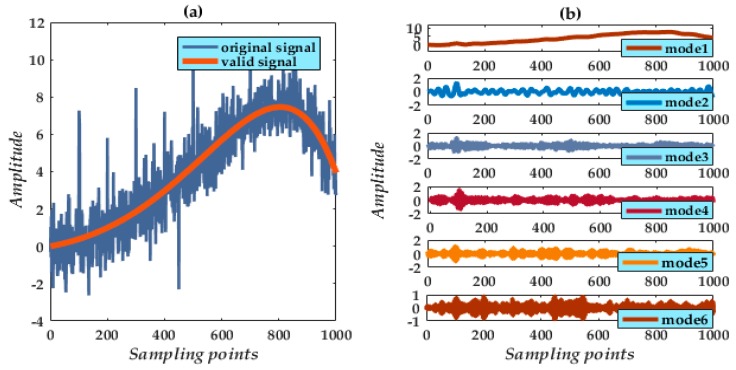
(**a**) Original and noisy simulated signals; (**b**) Six decomposed modes obtained using VMD.

In accordance with the compounded noise type, a combination SE (CSE) of triangle and semicircle is constructed. The waveform of the contaminated signal and the sampling points Figure 5a appear as a rough harmonic waveform. The original signal is completely immersed unable to be distinguished.

### 3.2. Signal Decomposition of the VMD

Through multiple adjustments, the sinusoidal signal produces excellent spectral characteristics from decomposition result by preestablishing the value *k* = 6. Various decomposition modes can be extracted Figure 5b, among which mode 1 is the apparent valid signal whereas modes 2 to 6 are high-frequency components identified as noise and need to be eliminated.

In general, valid signals have a distribution of high-frequency modes mixed in with the dominant mode. The objective of the present procedure is to remove directly the higher modes and retain the low-frequency mode as the main valid signal. However, this may lead to a loss of valid signal components. The retained signal may pose a distortion problem in lacking the finer details of the original signal. A final reconstruction can overcome this problem.

In determining SE lengths, RMSE values of the six modes are calculated to measure the degree of ambiguity. From Table 1, which lists actual calculated values, the PSE value of mode 1 is far below the other modes meaning a lower degree of ambiguity. From this indicator analysis, we obtain the corresponding SE lengths using the proposed numerical formulae Equations (21) and (22). In Table 1, a length of 1 from both TSE and SSE preserves mode 1 as having maximal degree in the CGMF processing. Applying the formulae, the TSE and SSE lengths for modes 2 to 6 are around 18 and 17 ± 2, respectively.

### 3.3. Application of the Multi-Scale CGMF

**Figure 6:**
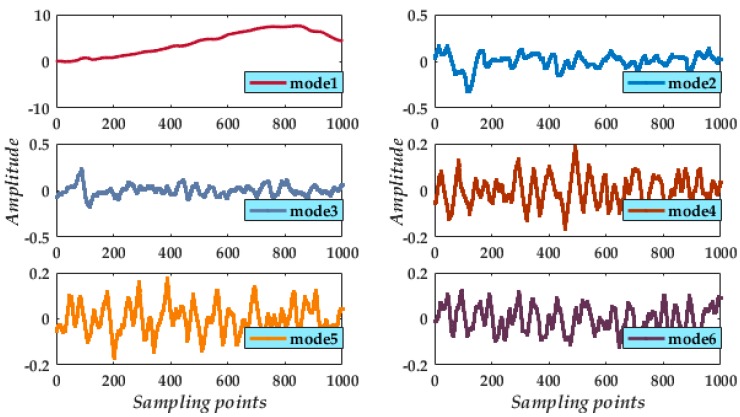
Waveforms of the preserved mode 1 and processed modes 2 to 6.

From the length formulae for each of the modes, the CGMF successively eliminates the contaminated noise. After de-noising the five modes and retaining mode 1 (Figure 6), the high-frequency noise is successfully suppressed, demonstrating that the indicators are of very practical use, and the remaining component is a valid component of the original signal.

**Figure 7:**
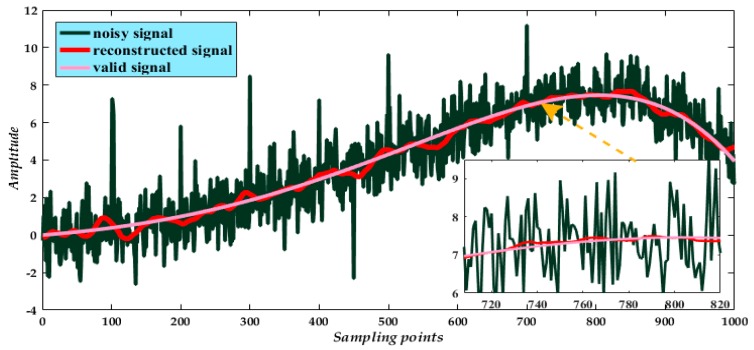
Final result of reconstructed signal.

Finally, from the reconstruction of mode 1 and processed modes (Figure 7), we compared the reconstructed signal with the valid signal and the noisy signal. We find the processed result attains the expected noise elimination. Indeed, from the partial enlarged segment of the signal (Figure 7, inset), the reconstructed signal has a high degree of overlap with the valid signal. Moreover, the whole reconstructed signal suitably reflects the instantaneous peaks in the noisy signal.

In Table 2, we added the corresponding noise of the different signal-to-noise power ratios (SNRs) to verify the noise reduction capability. Except for the above-mentioned filters, several currently effective de-noising methods are compared with our improved MF. Liu et al. [35] proposed the detrended fluctuation analysis (DFA)-VMD algorithm: a denoising method that combines VMD and a detrended fluctuation analysis (DFA). Cui et al. [31] developed the EMD-G-FLP algorithm: a hybrid filter. Both methods are designed for noise suppression in non-stationary vibration signals. Kang et al. [36] proposed an adaptive robust Kalman filter (ARKF)-based hybrid-correction grid strapdown inertial navigation system (SINS)/doppler velocity log (DVL) integrated navigation algorithm to improve the navigation accuracy. Thus, ARKF method is also applied for effect comparison.

**Figure 8:**
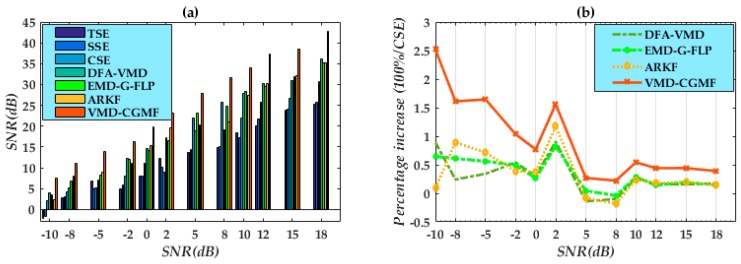
(**a**) SNRs of different algorithms for signal noise suppression; (**b**) incremental percentages of current algorithms compared to CSE method.

In comparing SSE, TSE, CSE, DFA-VMD, EMD-G-FLP, ARKF and CGMF-VMD methods, the SNRs of the denoising signal from CGMF-VMD are distinctively higher. The bars magnitude Figure 8a are larger than other methods. In order to facilitate comparison, different algorithms’ superiority analysis is based on CSE filter method. The incremental percentages Figure 8b of CGMF-VMD are superior to DFA-VMD and EMD-G-FLP to a large extend. Especially in heavy noise suppression with low SNRs, the proposed algorithm obtains better denoising performance than the other two. In global analysis, we comprehensively conclude that the CGMF-VMD method improved 57.31% in denoising, whereas DFA-VMD, EMD-G-FLP and ARKF improved 18.01%, 21.13% and 22.04% than CSE method respectively. Consequently, the parameter variations of the proposed indices enable an effective accuracy in extraction and efficacy in noise reduction. Digital simulations also demonstrate the SE length equation is feasible and the superior de-noising of the proposed method.

### 3.4. Computation Complexity of CGMF-Based on VMD Denoising Algorithm

To provide a comprehensive assessment of CGMF based on VMD algorithm, the algorithm is subjected to an analysis of computation time and space complexity. To simplify the analysis, we assume the time spent for all operators is the same and the computation complexity concerns only performance and running hardware. In regard to the complexity calculation, the operators involved all require estimating. Hence, we set up addition (ADD), subtraction (SUB), multiplication (MUL), definition (DEF), comparison (CMP) and division (DIV) [37].

We introduce *S* for the length of the input signal, i.e., the data scale, and *N* the maximum of loops and iterations. In the CGMF algorithm, the latter relates specifically to the open-close and close-open operator definitions of the actual SE construction. From the detailed computation, the time and space complexity corresponding to every procedure are listed in Table 3. The CGMF’s time and space complexity are both of linear order O (*N*).

Liu et al. [35] analyzed the VMD algorithm. Based on their work, the time and space complexity of the VMD algorithm is also shown in Table 4. The simulated signal is decomposed into k modes and the variate *N* in Table 4 denotes the maximum number of iterations. In our VMD algorithm, the initialization parameters are preset as follows: *alpha* = 2000*, tau* = 0, *tol* = 1×10^−7^, and *N* = 1000. From Table 4, the time and space complexity of VMD are of logarithmic order O ($2Nlog_{2}^{2N}$) and linear order O (*N*), respectively.

The time and space complexities for the CGMF based on the VMD algorithm are listed in Table 5. CGMF-VMD’s time complexity is of $O\;\left(2Nlog_{2}^{2N}\right)$ and the space complexity is of linear order O (*N*). According to the order of magnitude relation $O\;\left(N^{3}\right)>O\;\left(N^{2}\right)>O\;\left(2Nlog_{2}^{2N}\right)>O\;\left(N\right)>O\;\left(log_{2}^{2N}\right)>O\;\left(1\right)$, the results of the analysis demonstrate an improved MF based on the VMD method is a valid algorithm solvable in polynomial time. To compare the execution time of this algorithm with other algorithms, a simulation with signals of lengths ranging from 2^8^ to 2^16^ with SNR = 8 dB was performed on a personal computer (Intel^®^ Core™ 5@2.80 GHz and 8.00 GB RAM memory) running Windows 10.

In comparing EMD-G-FLP, DFA-VMD and ARKF algorithms, the same experimental conditions were applied in the tests. In the analysis of Yang et al., the time complexity for the EMD is $2Nlog2^{2N}$. The EMD-G-FLP and DFA-VMD algorithms’ time complexity are $O\;(2Nlog2^{2N}$), which are the same as CGMF-VMD. The ARKF algorithm has the largest magnitude of $O\;\left(N^{3}\right)$. The actual execution times are listed in Table 6. The ARKF execution time is maximum. The CSE complexity is least of O (N), execution times are the smallest, and CGMF-VMD is the second smallest through the factor influence of VMD. The execution times indicate that CGMF-VMD is still less than other two current methods, EMD-G-FLP and DFA-VMD. It can be explained that the influences of algorithm factors are different. The actual CGMF-VMD algorithm is with a smaller factor than G-FLP and DFA. From the previous analysis, CGMF-VMD execution times are slightly 9.87% more than CSE in Table 6, but 57.31% improvement in noise suppression effect. In addition, CGMF-VMD is 36% faster than ARKF, 14% faster than EMD-G-FLP and 6.0% faster than DFA-VMD. Thus, the proposed algorithm has a simple and rational construction as a whole.

## 4. Experimental Results and Analysis

### 4.1. Rotation Experimental Data Acquisition

**Figure 9:**
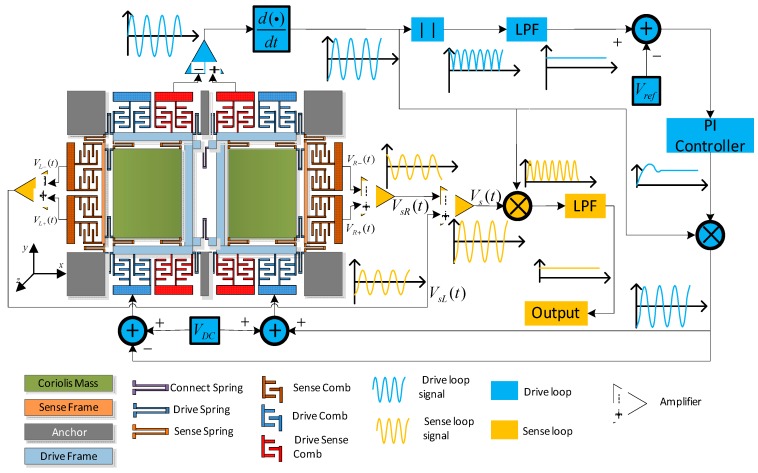
Schematic of the dual-mass decoupled MEMS gyroscope structure and periphery circuit.

The dual-mass decoupled gyroscope in the article is ceramic vacuum packaged. An AGC loop will stabilize the drive mode vibrating amplitude based on self-oscillation theory. In addition, the method ensures the drive mode works at its own resonant frequency. Gyroscope mechanical sensitivity achieves the maximum values with the same $A_{x}$. The sense loop utilizes open-loop method and phase sensitive demodulation technology, and a schematic of the gyroscope structure and peripheral circuits is shown in Figure 9 [38].

**Figure 10:**
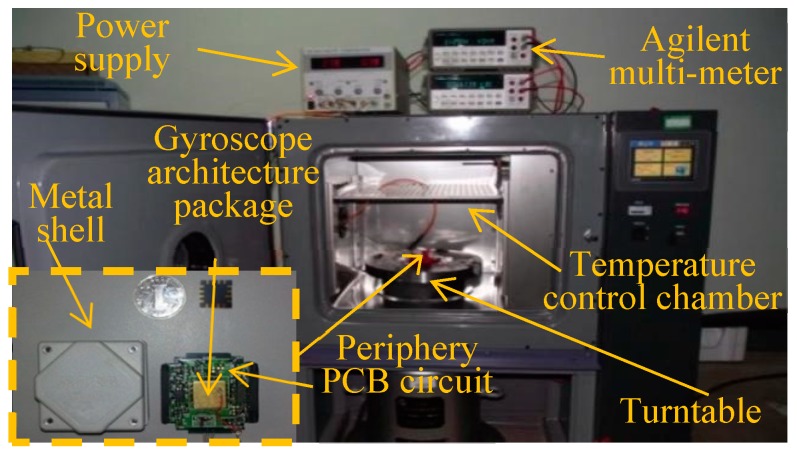
Test equipment for dual mass MEMS gyroscope.

**Figure 11:**
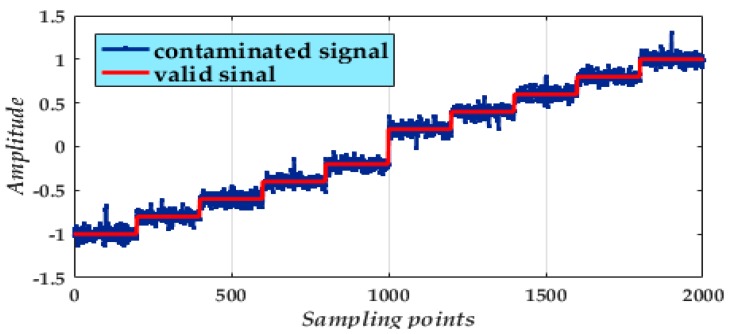
Experimental signal obtained from a rotating gyroscope.

In single mass MEMS gyroscope structures, sense axial accelerations often cause the invalidation of the sense signal. Nevertheless, A dual-mass structure method can suppress this phenomenon. To verify the effectiveness of our CGMF-VMD method, output signals collected from a dual-mass MEMS gyroscope is used in this experiment. The gyroscope was placed in a temperature-controlled oven (Figure 10). A digital multimeter (Agilent 34401A, Agilent technologies, Inc., Santa Clara, CA, USA) collected the output data from the gyroscope. Over an hour, one thousand sample points were recorded. A DC power supply (Agilent E3631A, Agilent Technologies, Santa Clara, CA, USA) provided an adjustable input power to a rotary table to preset rotation rates from −1°/s to +1°/s in incremental steps of 0.2°/s, each rotation rate corresponding to a sampling time of 200 s. The experimental signal obtained from the gyroscope (Figure 11) is mixed with heavy disturbances of impulse noises and Gaussian white noise. To eliminate the noise disturbance and generate stable characteristics of the gyroscope, noise suppression is necessary.

### 4.2. De-Noising Results and Comparisons

Based on the experimental noise characteristics, both TSE and SSE remain parallel-functional SEs. In the actual experimental noise reduction procedure, conventional filters using CSE MF, single TSE MF, and single SSE MF were applied for noise suppression test. After calculating related values evaluating the effect of noise reduction. We find the CSE MF method surely produces higher accuracy and effectivity than the conventional single filters. For CSE, both impulse noise and Gaussian white noise are simultaneously eliminated. However, the single filters SSE and TSE are only inclined to suppress noise of a single mode. The processed curve of SSE contains remains of an impulsive noise whereas for TSE Gaussian white noise has not been reduced significantly.

**Figure 12:**
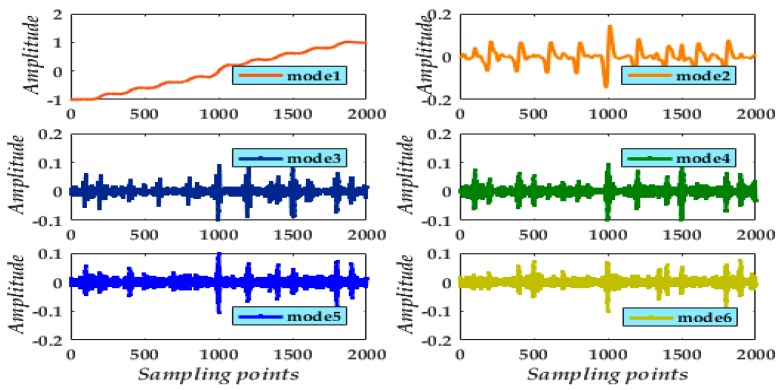
Results of decomposition for the experimental signal from a rotating gyroscope.

Applying the VMD method to the experimental signal and setting *k* = 6, the signals of six modes were extracted (Figure 12). We find mode 1 is primary in the gyroscope interference signal. Nevertheless, the preserved mode 1 poses a slight distortion and is displaced from the actual signal. Hence, applying de-noising to the other modes for reconstruction is essential. The experiment proceeded using the adaptive multi-scale CGMF combined with the VMD method to abate the problem and preserve a maximal valid signal.

Before determining the SE lengths, the PSE and RMSE values were calculated for all modes (Table 7). From the statistical multiple relationship between adjacent modes, TSE and SSE lengths were obtained using the length formulae, Equations (21) and (22). As dependent variables, the PSE parameter is highly relevant to the instantaneous state of SSE, and similarly RMSE to TSE. Finally, the scales of both SEs for each of the six modes are calculated (Table 7). To give further support to preserving mode 1, both length values of mode 1 return the best performances in the filtering. In comparison, the TSE and SSE length values for the other modes are around 33 ± 2 and 6 ± 1, respectively.

**Figure 13:**
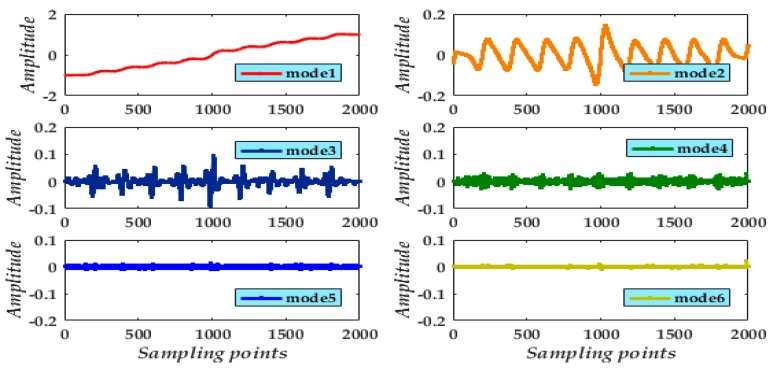
The persevered mode 1 and noise-suppression modes 2 to 6.

**Figure 14:**
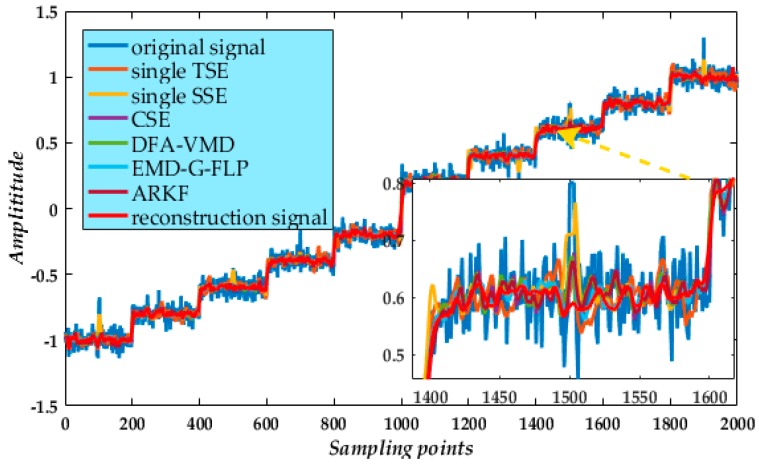
Comparison of the final reconstructed signal from various de-noising methods.

With these calculated SE lengths, noise suppression results for modes 2 to 6 using the adaptive multi-scale CGMF (Figure 13) show the high-frequency noise are eliminated. The valid low-frequency signal includes modes 2 and 3 and is accurately extracted, the waveform being relatively smooth. Finally, the reconstructed signal from the gyroscope with the six modes (Figure 14) enables a detailed comparison of the effect of noise suppression for the different types of filters.

Under evaluation values analysis In Table 8, we find the CSE, single TSE, and single SSE (Figure 14) method had poor performance. Specifically, the denoised waveforms obtained from the DFA-VMD, EMD-G-FLP and ARKF methods (Figure 14) are compared with the reconstructed signal from CGMF-VMD method. In Table 8, the RMSE, PSE, and standard deviation (STD) values of the three current algorithms and our method are listed. From the noise reduction waveforms and the indicator analysis, the result proves that the improved MF combined with the VMD algorithm is more effective. The reconstructed result is compared with the original experimental signal. Clear noise suppression is evident proving that the length formulae Equations (21) and (22) are viable. Compared with all other waveforms, the reconstructed waveform of the gyroscope signal is smooth and the heavy noise is eliminated. Moreover, there is less interference. Hence, the improved CGMF-VMD method in gyroscope de-noising yields a better valid signal than conventional filter methods and preserves mode content. The de-noising result of ARKF has a fine de-noising performance but is not as smooth as CGMF-VMD method, that is because the ARKF is de-noising the signal in time-domain directly, while the proposed method denoise different BLIMFs independently which makes the de-noising process more specific.

To provide quantitative comparison about angle random walk (ARW) and bias instability values, Allan variance analysis can reflect the de-noising results of different methods. *Q* (quantification noise), *N* (ARW), *B* (bias instability), *K* (rate random walk) and *R* (angular rate ramp) are five random noise coefficients respectively in Allan variance analysis for a dull-mass MEMS gyroscope. From the coefficients values analysis in Table 9, the proposed algorithm has a better performance in ARW and bias instability, which shows better advantage in gyroscope de-noising.

### 4.3. Results Analysis and Consideration

After applying the adaptive multi-scale CGMF-VMD method, the final gyroscope reconstructed waveform yields a satisfactory de-noising. The indicator values for RMSE, PSE and STD (Table 8) show that this method eliminates the various types of noise more thoroughly than other conventional filters. The Allan variance results (Table 9) also demonstrate the superiority of this method in gyroscope application.

Nevertheless, there are some significant issues to consider regarding the analysis of actual experiment results. High-intensity mixed noise is significantly different. Choosing an appropriate SE combination, each of which suppresses specific noise types, is a skilled procedure. The critical step is to properly pick a real-time indicator in assessing inhibiting effects of SE. As for length selection, the author proposes preferred formulae for PSE and RMSE, which experimental results have demonstrated are practical over numerous platform data analysis.

In the VMD, the selection of the value for *k* is a priori knowledge to be determined. For achieving more thorough stratified noise suppression, the decomposed modes should possess manageable features for de-noising processing. In resolving the SE combination, indicator selection, and length determining problem, the proposed method appears highly practical and reliable.

## 5. Conclusions

An innovative approach is proposed for noise elimination in gyroscope signals. The proposed adaptive multi-scale CGMF based on the VMD method is distinctly better than previous de-noising approaches. CGMF is applied to the gyroscope’s output signal for noise reduction. The VMD is called within the CGMF to improve its performance.

For determining SE length, PSE and RMSE are indicators that reliably assess TSE and SSE performance, respectively. Also, extensive experiments were conducted to prove the feasibility of the proposed selection formulae based on the indicators. With the analyses of different types of noise to be reduced, a gyroscope experiment provided evidence for the effectiveness of the proposed algorithm in an application to an output signal with both heavy impulsive noise and random white noise.

In the comparison to current algorithms, the actual time complexity of proposed method is 6–14% less, and the denoising effect can improve about 35% more. Thus, we can conclude that our proposed method is feasible and superior in noise suppression filed.

## Figures and Tables

**Table 1:** Numerical results for the different modes: RMSE and PSE values and calculated lengths corresponding to each mode and SE.

Mode	Parameter Values		Corresponding Scale	
	RMSE	PSE	TSE	SSE
Mode1	1.13	0.45	1	1
Mode2	4.83	1.73	18	15
Mode3	4.82	1.69	18	14
Mode4	4.83	1.79	18	16
Mode5	4.82	1.84	17	17
Mode6	4.83	1.92	18	19

**Table 2:** Comparison of the adaptive multi-scale CGMF with other conventional filters denoising.

**SNR (dB)**	−10	−8	−5	−2	0	2	5	8	10	12	15	18
**TSE**	−2.20	2.84	6.78	5.03	7.94	12.19	13.76	14.93	18.45	20.13	23.89	25.14
**SSE**	−1.64	3.14	4.99	5.94	8.03	10.21	14.35	15.16	17.31	21.70	24.13	25.64
**CSE**	2.12	4.21	5.26	8.03	11.17	9.03	22.01	22.79	22.01	25.84	26.71	30.81
**DFA-VMD**	4.01	5.23	7.06	12.36	14.56	17.23	18.92	23.22	27.90	30.14	30.89	36.23
**EMD-G-FLP**	3.49	6.78	8.21	11.99	14.12	16.48	23.04	24.76	28.34	29.45	31.97	35.14
**ARKF**	2.31	7.97	9.01	11.17	15.34	19.65	20.34	21.08	27.31	30.31	32.10	35.28
**VMD-CGMF**	7.46	10.99	13.91	16.34	19.77	23.13	27.94	31.53	33.95	37.23	38.51	42.83

**Table 3:** Evaluating time and space complexity for adaptive multi-scale CGMF.

Function	T	M
**Initialize**	5DEF·S	[4 + S] float
**B1 SE Construction**	(4DEF + 1CMP)·S·*N* + 1DEF	[5*N*] float
**B2 SE Construction**	(3DEF + 1ADD + 3SUB + 1DIV + 2DEF)·S·*N*	[12*N* + 6]·S float
**Operator Run**	(4DEF + 4ADD + 4DIV + 4CMP)·S	[4*N·*S] float
**CGMF Basic Arithmetic Unit**	(1ADD + 1DIV)·S	[2S] float
**Complexity**	O (N)	O (*N*)

**Table 4:** Evaluating time and space complexity for the VMD algorithm.

Function	T	M
**Initialize**	O ($\;2Nlog_{2}^{2N})$	[(3 + K·*N* + *N*)·2S + K·*N*] float
**Update** $u_{k}$	(6ADD + 2MUL + 2DIV)·K·*N*·2S	[2S] float
**Update** $w_{k}$	(2CMP + 3MUL + 2ADD)·K·*N*·S	0
**Dual Ascent**	(4ADD + 1MUL)·*N*·2S	0
**Convergence**	(4ADD + 2MUL)·*N*·2S	0
**Complexity**	O ($2Nlog_{2}^{2N})$	O (*N*)

**Table 5:** Time and space complexity for the improved MF based on the VMD algorithm.

Function	T	M
**CGMF**	O (N)	[(16*N* + 9)·S + 5*N* + 4] float
**VMD**	O ($\;2Nlog_{2}^{2N})$	[(4 + K·*N* + *N*)·2S + K·*N*] float
**CGMF based on VMD**	O ($\;2Nlog_{2}^{2N})$ + (K + 1)·O (N)+$k^{\prime }$·1ADD·S	[(4 + K·*N* + *N*) ·2S + K·*N*] + (K + 1)·[(16*N* + 9)·S + 5*N* + 4] + S float
**Complexity**	O ($2Nlog_{2}^{2N})$	O (*N*)

**Table 6:** Execution times for a simulated signal applying different noise suppression methods.

*n*	EMD-G-FLP	DFA-VMD	CSE	ARKF	CGMF-VMD
$2^{8}$	0.1918	0.1934	0.1783	0.2098	0.1813
$2^{9}$	0.3784	0.3516	0.2716	0.6832	0.3125
$2^{10}$	0.6347	0.5927	0.4917	0.9246	0.5482
$2^{11}$	1.9672	1.3638	1.2588	2.2351	1.3094
$2^{12}$	1.8981	1.4824	1.2763	2.8735	1.3803
$2^{13}$	3.4597	3.1249	2.8081	8.8931	3.0341
$2^{14}$	7.0560	6.8691	6.2113	14.3721	6.4283
$2^{15}$	28.4978	25.2678	20.2587	31.8724	22.4589
$2^{16}$	47.3541	46.1564	39.5411	63.1963	44.5622

**Table 7:** Experimental calculated data for each decomposed mode: values from indicators RMSE and PSE, and calculated lengths of TSE and SSE.

Mode	Parameter Values		Corresponding Scale	
	RMSE	PSE	TSE	SSE
**Mode 1**	0.12	0.92	1	1
**Mode 2**	0.67	2.00	31	5
**Mode 3**	0.66	2.20	30	6
**Mode 4**	0.69	2.17	33	6
**Mode 5**	0.70	2.30	34	7
**Mode 6**	0.71	2.24	35	7

**Table 8:** Results from an experimental evaluation comparing indicator values of RMSE, PSE and STD of the original signal with those from different noise reduction methods.

Nosie Reduction Methods	RMSE	PSE	STD
Original Signal	0.0931	0.959	0.865
CSE	0.0590	0.633	0.614
Single SSE	0.0709	0.737	0.785
Single TSE	0.0757	0.694	0.681
DFA-VMD	0.0430	0.431	0.439
EMD-G-FLP	0.0413	0.393	0.456
ARKF	0.0274	0.348	0.267
Experimental Reconstruction Signal	0.0208	0.192	0.201

**Table 9:** Allan variance analysis results before and after de-noising of MEMS gyroscope.

Noise Reduction Methods	*Q* (μ rad)	*N* (°/h^1/2^)	*B* (°/h)	*K* (°/h^3/2^)	*R* (°/h^2^)
Original Signal	5.764	0.408	11.329	6.76	4.64
CSE	3.766	0.304	10.330	6.36	4.62
Single SSE	4.239	0.388	11.021	6.28	4.63
Single TSE	2.654	0.291	10.829	6.37	4.61
DFA-VMD	2.071	0.279	9.331	6.58	4.61
EMD-G-FLP	1.768	0.263	9.535	6.29	4.62
ARKF	1.973	0.227	9.336	6.28	4.63
Experimental Reconstruction Signal	1.672	0.203	9.017	6.27	4.61
